# Toward an Improved Understanding of the Ingestion and Trophic Transfer of Microplastic Particles: Critical Review and Implications for Future Research

**DOI:** 10.1002/etc.4718

**Published:** 2020-05-22

**Authors:** Todd Gouin

**Affiliations:** ^1^ TG Environmental Research, Sharnbrook, Bedfordshire United Kingdom

**Keywords:** Microplastic, Bioaccumulation, Exposure, Environmental fate

## Abstract

Microplastic particles have been observed in the environment and routinely detected in the stomachs and intestines of aquatic organisms over the last 50 yr. In the present review, information on the ingestion of plastic debris of varying sizes is collated, including data for >800 species representing approximately 87 000 individual organisms, for which plastic debris and microplastic particles have been observed in approximately 17 500, or 20%. The average reported number of microplastic particles/individual across all studies is estimated to be 4, with studies typically reporting averages ranging from 0 to 10 particles/individual. A general observation is that although strong evidence exists for the biological ingestion of microplastic particles, they do not bioaccumulate and do not appear to be subject to biomagnification as a result of trophic transfer through food webs, with >99% of observations from field‐based studies reporting that microplastic particles are located within the gastrointestinal tract. Overall, there is substantial heterogeneity in how samples are collected, processed, analyzed, and reported, causing significant challenges in attempting to assess temporal and spatial trends or helping to inform a mechanistic understanding. Nevertheless, several studies suggest that the characteristics of microplastic particles ingested by organisms are generally representative of plastic debris in the vicinity where individuals are collected. Monitoring of spatial and temporal trends of ingested microplastic particles could thus potentially be useful in assessing mitigation efforts aimed at reducing the emission of plastic and microplastic particles to the environment. The development and application of standardized analytical methods are urgently needed to better understand spatial and temporal trends. *Environ Toxicol Chem* 2020;39:1119–1137. © 2020 The Authors. *Environmental Toxicology and Chemistry* published by Wiley Periodicals LLC on behalf of SETAC.

## INTRODUCTION

The ingestion of contaminants that have the potential to bioaccumulate and biomagnify in organisms at various trophic levels in food webs is an important component of chemical regulatory activity in several jurisdictions (Gobas et al. 2009; Swackhammer et al. 2009; Schwarzenbach et al. 2017). Characterization and quantification of the bioaccumulation potential of chemical contaminants, for instance, are largely based on the wealth of data that exist in relation to our understanding of the environmental fate of persistent hydrophobic organic chemicals in aquatic environments. Briefly, the underlying theory for the quantification of bioaccumulation for an aqueous exposure relies on characterization of the concentration ratio at steady state between the water (*C*_W_ as mg/L) and the tissue concentration of the organism, typically fish (*C*_f_ as mg/kg), to derive a bioconcentration factor, expressed as L/kg (Gobas et al. 2009; Schwarzenbach et al. 2017), when exposure is only via the external medium, in this instance water. Alternatively, a biomagnification factor can also be characterized, which would include both respiratory uptake and dietary exposure.

For chemical contaminants in aqueous systems, exposure by either respiration or ingestion and subsequent biological uptake and accumulation in the tissues of aquatic organisms is driven by a thermodynamic energy gradient that inevitably dissipates, giving rise to a steady‐state concentration ratio between the organism and its external environment. A simple 2‐compartment model can be defined, in which the net flux of the chemical into the organism (i.e., accumulation) is characterized by the sum of the fluxes for all uptake mechanisms or loss processes, which can be derived from the ratio of uptake (*k*_1_) and elimination (*k*_2_) rate constants (Gobas and Morrison 2000; Schwarzenbach et al. 2017). Typically, assessment of the bioaccumulation and quantification of *k*_1_ and *k*_2_ is achieved by conducting an in vivo study following Organisation for Economic Co‐operation and Development (2012) test guideline 305, which has recently been revised to include a protocol for including dietary uptake.

Concerns related to the potential for bioaccumulation are not necessarily limited to chemical contaminants but have recently been raised with respect to poorly soluble particulates such as engineered nanomaterials (ENMs; Hou et al. 2013; Martirosyan and Schneider 2014; Lead et al. 2018; Petersen et al. 2019) and microplastic particles (Watts et al. 2014; Van Cauwenberghe et al. 2015; Karlsson et al. 2017; Barboza et al. 2018; Carbery et al. 2018; Dawson et al. 2018). A fundamental challenge in assessing the bioaccumulation of poorly soluble particulates is that the accumulation process is not driven by thermodynamic energy gradients, but by physical processes (Petersen et al. 2019). For soluble chemicals, bioaccumulation can be perceived as a pseudo‐intrinsic property (Mackay et al. 2001), and is thus not dependent on parameters such as external concentration. The environmental fate and behavior of poorly soluble particulates, on the other hand, is influenced by the relative particle number concentration, which is subsequently influenced by the collision frequency and energy in aqueous systems (Handy et al. 2018). The interaction between organisms and particulates such as ENMs and microplastic particles is thus dynamic, not steady state, and the processes for uptake and elimination are driven by various endocytosis‐related mechanisms (Felix et al. 2017), not by passive diffusion through tissues or via solute transporters as they are for soluble chemicals (Schultz 1976; DeVito 2000). Consequently, the assessment of poorly soluble particulates with respect to their potential to bioaccumulate may require the development of new test systems, models, and mechanistic understanding (Handy et al. 2018; European Centre for Ecotoxicology and Toxicology of Chemicals 2019; Petersen et al. 2019; Roch et al. 2020).

Nevertheless, in the absence of tools applicable to assessment of the bioaccumulation of poorly soluble particulates, concerns regarding their ingestion and potential to bioaccumulate, particularly for microplastic particles, have recently been growing. For instance, both laboratory‐ and field‐based studies have aimed directly at assessing the extent and mechanisms related to the ingestion and trophic transfer of microplastic particles (Farrell and Nelson 2013; Santana et al. 2017; Chae et al. 2018; Chagnon et al. 2018; Macali et al. 2018; Nelms et al. 2018; Welden et al. 2018; Zhao et al. 2018). These studies have demonstrated that organisms at lower trophic levels, such as macrozooplankton, are capable of ingesting microplastic particles either indirectly or directly as a consequence of mistaking the particles for food, and are then themselves ingested by organisms at higher trophic levels, suggesting a potential mechanism that might support arguments for the bioaccumulation and biomagnification of microplastic particles. A common theme in these studies, however, is that the microplastic particles themselves have been observed throughout the gastrointestinal tract, with the particles subsequently egested following a depuration period (von Moos et al. 2012; Au et al. 2015; Grigorakis et al. 2017; Santana et al. 2017; Dawson et al. 2018; Cong et al. 2019; Fernandez and Albentosa 2019). In some studies the potential for translocation of the particles to internal tissues has been observed and reported (Hussain et al. 2001; Browne et al. 2008; von Moos et al. 2012; Avio et al. 2015; Abbasi et al. 2018; Ding et al. 2018). Caution has been suggested, however, regarding the potential for translocation of microplastic particles based on observations utilizing fluorescence, because the results may be characteristic of a study artifact (i.e., lipid accumulation of the leached fraction of hydrophobic fluorescent dye), as opposed to actual particle translocation (Schur et al. 2019). Consequently, quantifying and characterizing the bioaccumulation potential of microplastic particles within the tissues of organisms would benefit from the adoption of a mass balance approach, aimed at assessing adsorption, distribution, metabolism, and excretion (Gobas and Morrison 2000; Al‐Sid‐Cheikh et al. 2018; Diepens and Koelmans 2018; Petersen et al. 2019).

Given the relative efficiency of the egestion of microplastic particles that has been observed and reported, the relative difference between uptake/adsorption and loss/excretion is likely to be the key parameter requiring quantification, assuming metabolism and internal distribution to be relatively negligible in comparison. Thus, from the perspective of assessing bioaccumulation, it remains largely unclear whether the accumulation of microplastic particles within the tissues of an organism exceeds the concentration of such particles in the surrounding environment—a fundamental characteristic for defining bioaccumulation. Alternatively, the observations of microplastic particles within the gastrointestinal tract of organisms may more appropriately represent dynamic snapshots in time and space, information that may inform on the relative level of particle contaminants.

The purpose of the present study was thus to review the existing literature with respect to the ingestion, bioaccumulation, and trophic transfer of microplastic particles and to critically evaluate the weight‐of‐evidence that might support or refute the potential for microplastic particles to bioaccumulate. In other words: 1) Are the concentrations of microplastic particles in organisms ≫ the concentration of the surrounding environment? and 2) Are the concentrations of microplastic particles in organisms at higher trophic levels ≫ the concentrations in their prey?

Additional objectives were to identify spatial and temporal trends, summarize existing knowledge gaps, and, based on an overall evaluation of the data, provide guidance toward future research and determine the implications with respect to interpreting data reporting on the ingestion of microplastic particles.

## MATERIALS AND METHODS

### Literature review

The literature data on ingestion of microplastic particles to include in the present review was initially based on the reference lists reported in several robust reviews recently published for organisms at all levels of biological organization (Schuyler et al. 2014; Deudero and Alomar 2015; Lusher et al. 2017; Poeta et al. 2017; Burns and Boxall 2018; Foley et al. 2018; Fossi et al. 2018; Hermsen et al. 2018; Battisti et al. 2019; Botterell et al. 2019; Triebskorn et al. 2019; Wang et al. 2019). The initial list was further complemented by identification of other studies, using a range of methods. These included keyword searches of “microplastics” and/or “bioaccumulation,” “ingestion,” and “trophic transfer” using various sources, such as Google Scholar, ResearchGate, and Scopus. In all cases, further attention was also given to the reference lists included in all additional studies that were added; in addition, newer publications were identified by tracing citation lists. The objective of the approach adopted was to develop a comprehensive dataset to assess the biological ingestion, bioaccumulation, and trophic transfer of microplastic particles and other plastic debris by organisms at all levels of biological organization that have been documented since the mass production of plastic was introduced to society. An important element of the process for assembling studies is a recognition that the use of terms such as “microplastic,” “microplastics,” and “micro‐plastic” is relatively new in the peer‐reviewed literature. For several decades, a number of studies have documented the observation of ingestion and/or interactions with plastic debris by organisms; these would not necessarily be captured if one relied solely on a keyword search. The challenge associated with the development of new terminology and shifting awareness in undertaking this literature review is thus acknowledged to be laborious and potentially difficult to replicate, but the extent of the number and diversity of studies included in the analysis should provide beneficial insights into both spatial and temporal trends and should also illuminate some implications for future research activities.

Defining which types of particles can best be characterized as microplastic particles remains a topic of current debate (Verschoor 2015; Kramm et al. 2018; Frias and Nash 2019; Rochman et al. 2019). As generally defined, microplastic particles are synthetic plastic particles <5 mm in size. However, studies included in the present review have targeted the ingestion of both microplastic particles and plastic debris, for which size ranges may be above or below the <5‐mm size. In this instance, studies that specifically reported on the ingestion of either microplastic particles or simply plastic have been included, with extraction of the following information from each study (when given): the species studied; the number of individuals included; the location where individuals were collected and year of collection; the number of individuals containing microplastic particles; the maximum, minimum, average, and standard deviation of the number of particles/individual; the maximum, minimum, and average mass of the particles; the size of the particles observed; the shape and polymer composition of the particles; the location within the organism where the particles were observed; the methods used for sample collection and processing; the analytical tools used to identify the particles; the measures taken to reduce airborne contamination; and any other pertinent information related to biological ingestion, bioaccumulation, and trophic transfer. For laboratory‐based studies, details regarding the exposure conditions, specifically exposure dose and duration, were extracted.

### Data analysis

Various approaches toward analysis of the assembled dataset were used. These included an assessment of the quality of each of the field‐based studies as reported for individual species based on the quality criteria and assessment scoring system developed and applied by Hermsen et al. (2018). It should be noted that data on the number of microplastic particles/individual are generally perceived as being the most relevant for understanding the ingestion and subsequent potential for ingestion and also for enabling a comparison between organisms of different genders, ages, and species. Consequently, data on microplastic particles/individual were prioritized in assessing trends in space and time.

The quality assessment method developed was based on 10 separate criteria: sampling method and strategy; sample size, processing, and storage; laboratory preparation; clean air conditions; adoption of negative and positive controls; target component; sample treatment both before and after a procedure; and polymer identification. Each criterion was assigned a score of either 0, 1, or 2, with 0 indicating that the criterion was not adequately addressed, 1 suggesting the criterion was met but with caveats, and 2 implying the criterion was adequately addressed (Hermsen et al. 2018). A total assessment score (TAS) was then derived by summing the score for each of the individual criteria. Although Hermsen et al. (2018) provide guidance on how to score a study for each criterion, there is the potential for subjectivity in some instances, whereby assigning a 1 or a 2 may be open to interpretation. In recognition of this potential challenge, the results of the study assessments were binned to reflect more general trends in the TAS, with a TAS ≤10 implying that a study largely failed to meet the quality of the defined criteria, a TAS between 10 and 15 indicating that study results were of average quality, and a score ≥15 implying that the study had satisfactorily addressed each of the specific criteria defined.

To assess trophic transfer and the potential for biomagnification of microplastic particles through the food web, the relative trophic levels for each species were obtained from various sources (FishBase; SeaLifeBase; other relevant studies reporting species‐specific trophic levels; Hobson 1993; Hobson et al. 1994; Cherel et al. 2010; Lavoie et al. 2010; Pasquaud et al. 2010; Morkūnė 2011); then the relationships between the number of microplastic particles/individual (when reported) as well as the frequency of detection/species (when reported) and trophic level were assessed. A species trophic position represents a quantitative measure of its relative energetic interactions and is one of the most widely used descriptors of the role of a species within an ecological community (Carscallen et al. 2012). The specific trophic position has been correlated with a variety of factors, such as variation in body size, consumer‐resource size ratio, species range, interaction strength, distribution of energy flow in food webs, and bioaccumulation. The trophic levels span a range of 1 to 5, reflecting different positions within an ecological pyramid: primary producers are at the base of the pyramid (trophic level of ~1), apex predators are at the top (trophic level approaching 5), and primary, secondary, and tertiary consumers respectively represented by increasing tropic levels in between.

Analysis of the data also included an assessment of the effectiveness of a fish species for use as a biomonitor, which is determined using 5 criteria defined by Bray et al. (2019) in their formulation of a bioindicator index. The 5 criteria include the distribution of a species on a global scale, the length of the gastrointestinal tract, the commercial value, the home range and vagility, and the observed occurrence (%) of plastic reported in the literature for each species. Each of the 5 factors is assigned a ranking score, and the average of all ranked scores is used in estimating the bioindicator index. Given the criterion that emphasizes the relative importance of the commercial value of a fish species, only those species included in the present review with known commercial value were used for assessing a bioindicator index. This includes the assessment of >30 fish and seafood species, with data on their commercial value obtained from a European Market Observatory for Fisheries and Aquaculture Products (2018) report. Additional details are provided in the Supplemental Data.

## RESULTS AND DISCUSSION

### Literature review

**Figure 1:**
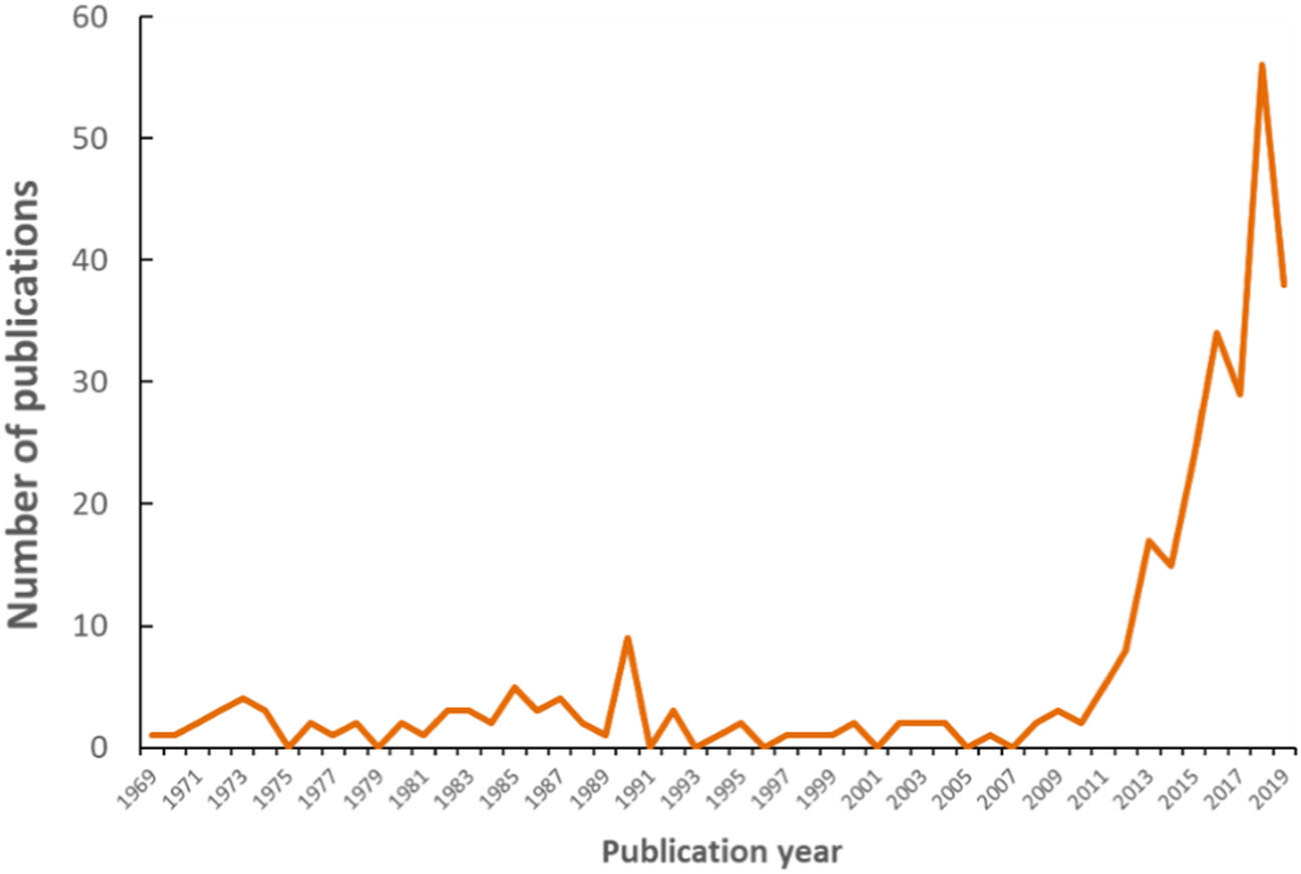
Plot illustrating exponential increase over time of number of publications reporting on the biological ingestion of plastic debris and microplastic particles.

Based on the method adopted to assemble relevant studies on the ingestion of microplastic particles, a total of 421 individual studies was comprehensively reviewed (Supplemental Data). Of the 421 studies, 305 reported data obtained from either field‐based (226/305) or laboratory‐based observations (79/305). The studies cover the period between 1929 and 2019. Figure 1 illustrates that particularly over the last decade, specifically between 2009 and the end of June 2019, there has been an exponential increase in the number of studies reporting the ingestion of plastic by organisms at all levels of biological organization. Whereas the research and documentation of ingestion by organisms represents an emerging concern, the actual interaction with plastic litter does not necessarily represent an “emerging” hazard. The hazard associated with plastic litter has been present for several decades, as implied from observations of harmful interactions as early as 1929 (Gudger 1929), but recent interest appears to be associated with an emerging collective societal awareness.

**Figure 2:**
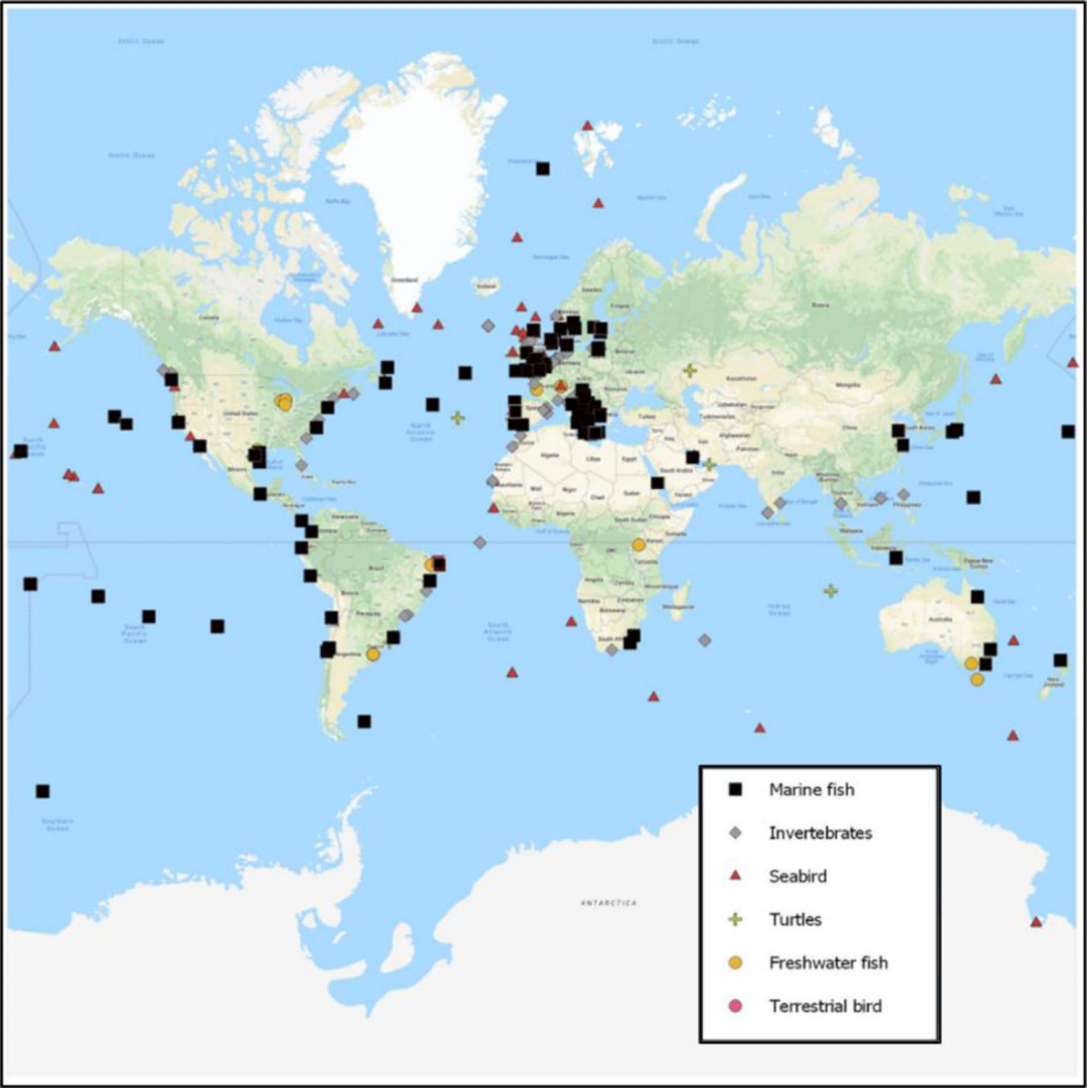
Relative geographic locations of studies included in the present review and broad categories of organisms studied.

With respect to the extent of analyses conducted thus far, 87 000 individual organisms have been sampled from field‐based studies assessing the ingestion of microplastic particles or other larger items of plastic debris. The total number of individuals for which data reporting on ingestion of microplastic particles and/or larger items of plastic debris was 17 500, or approximately 20% of the 87 000. The data represent vast geographic locations, covering all oceans and continents, as illustrated in Figure 2. Both marine and freshwater systems are included as well as terrestrial organisms. As seen in Figure 2, some regions have been more heavily studied than others, with marine fish and invertebrates collected from European coastal waters representing 30% of all data. In the 305 studies reporting data from either field‐ or laboratory‐based studies, >800 species have been assessed. Given the extent of species and numbers of individual organisms studied, the database assembled (Supplemental Data) supports observations that ingestion of microplastic particles occurs at all levels of biological organization, globally, with observations spanning several decades. It should also be noted, however, that observations of ingestion are highly variable, both spatially and temporally, suggesting caution when interpreting potential relationships associated with ingestion.

### Microplastic size, shape, and characteristics

Data on the physical characteristics of microplastic particles can potentially help to identify sources, information that can then help guide mitigation efforts. A key challenge, however, relates to limitations in analytical capability. Several reviews summarizing the advantages and disadvantages of the various analytical methods have been published (Hidalgo‐Ruz et al. 2012; Rocha‐Santos and Duarte 2015; Lusher et al. 2017; Hermsen et al. 2018). There is clearly a need for standardized quantitative methods. In the absence of standardized methods, characterization of microplastic particles is thus limited to the analytical capability of the individual laboratory performing the analysis. Variability in the quality of data produced is substantive. Some information is reported using “citizen science,” which relies on visual identification under the microscope. Other studies have employed advanced techniques such as Fourier transform infrared (FTIR) and/or Raman spectroscopy to verify the polymer composition of microplastic particles. Nevertheless, regardless of the level of analytical sophistication used in deriving these data, the information obtained can be used qualitatively to gain insights into spatial and temporal trends as well as prioritization for developing appropriate standardized analytical methods.

**Figure 3:**
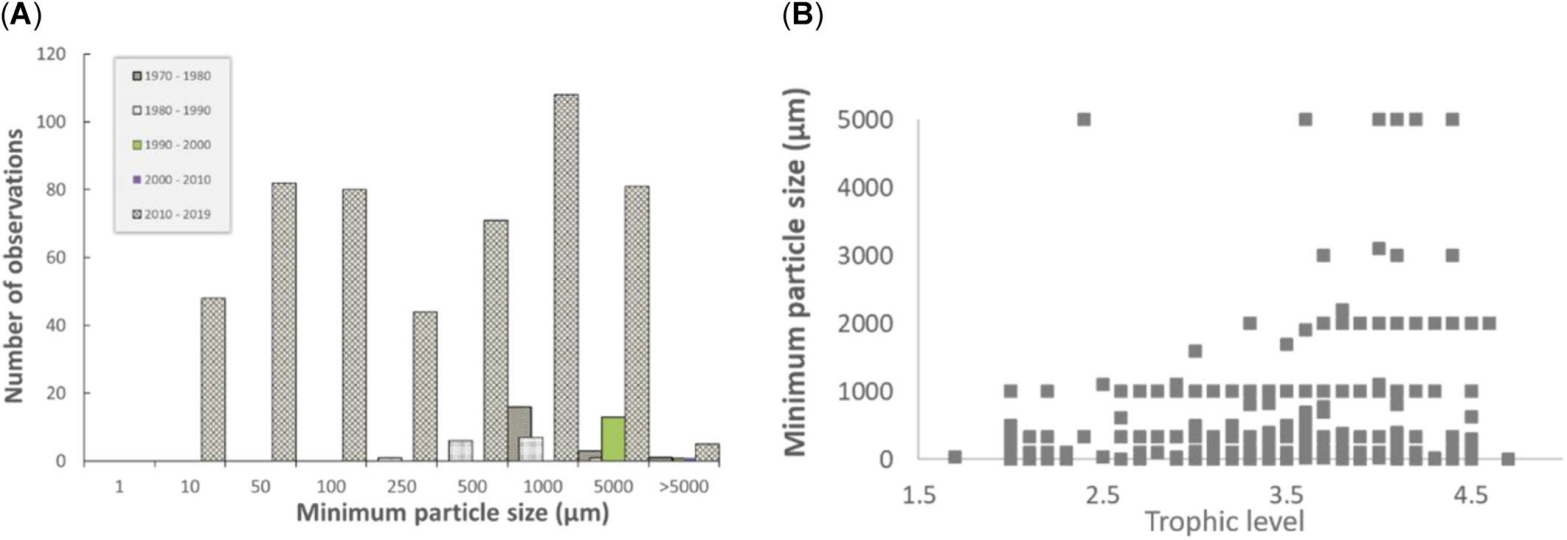
Summary of (**A**) particle size distribution for studies characterizing and quantifying the particle sizes of microplastic particles ingested by biological organisms and (**B**) minimum particle sizes ingested by organisms at varying trophic levels.

For instance, Figure 3 illustrates the distribution of the minimum particle sizes for which data are reported in relation to the ingestion of microplastic particles by organisms. Particle sizes >0.5 mm has tended to be the predominant size‐fraction of particles reported. The reporting of particle sizes, however, represents a relatively recent trend, and a decrease in the minimum size of particles has been described since 2010 (Figure 3A), prior to which the ability to observe particles <0.25 mm was generally absent. With improvements in analytical capability and quality control, there has thus been a relatively recent shift in the reporting of particle size to better assess the abundance of particles ingested that are <0.25 mm. This is an important development, particularly given the speculation that environmental exposure to microplastic particles down to the nanosize potentially represents a greater hazard to organisms than the size‐fractions that have typically been reported thus far (Gregory 2009; Andrady 2015; Barboza et al. 2018). However, it should be noted that the data shown in Figure 3 do not appear to reflect a higher frequency of minimum particle sizes <0.25 mm, but rather a relatively uniform distribution for particle sizes ranging from 0.01 to 1 mm. Care should thus be taken when one is attempting to interpret the size distribution of microplastic particles ingested by organisms, because changes in analytical ability over time have resulted in a shift in particle size distributions reported, which are not necessarily reflective of reality. This change in analytical capability has resulted in inconsistencies regarding the minimum particle size ingested by organisms, making it difficult to interpret trends regarding potential relationships between particle sizes ingested by organisms at varying trophic levels (Figure 3B). To increase the ability to assess temporal trends and better understand species‐specific differences in relation to particle size ingestion, it will be necessary to establish standardized methods that could be used to characterize and quantify a baseline, from which data in the future can be compared.

**Figure 4:**
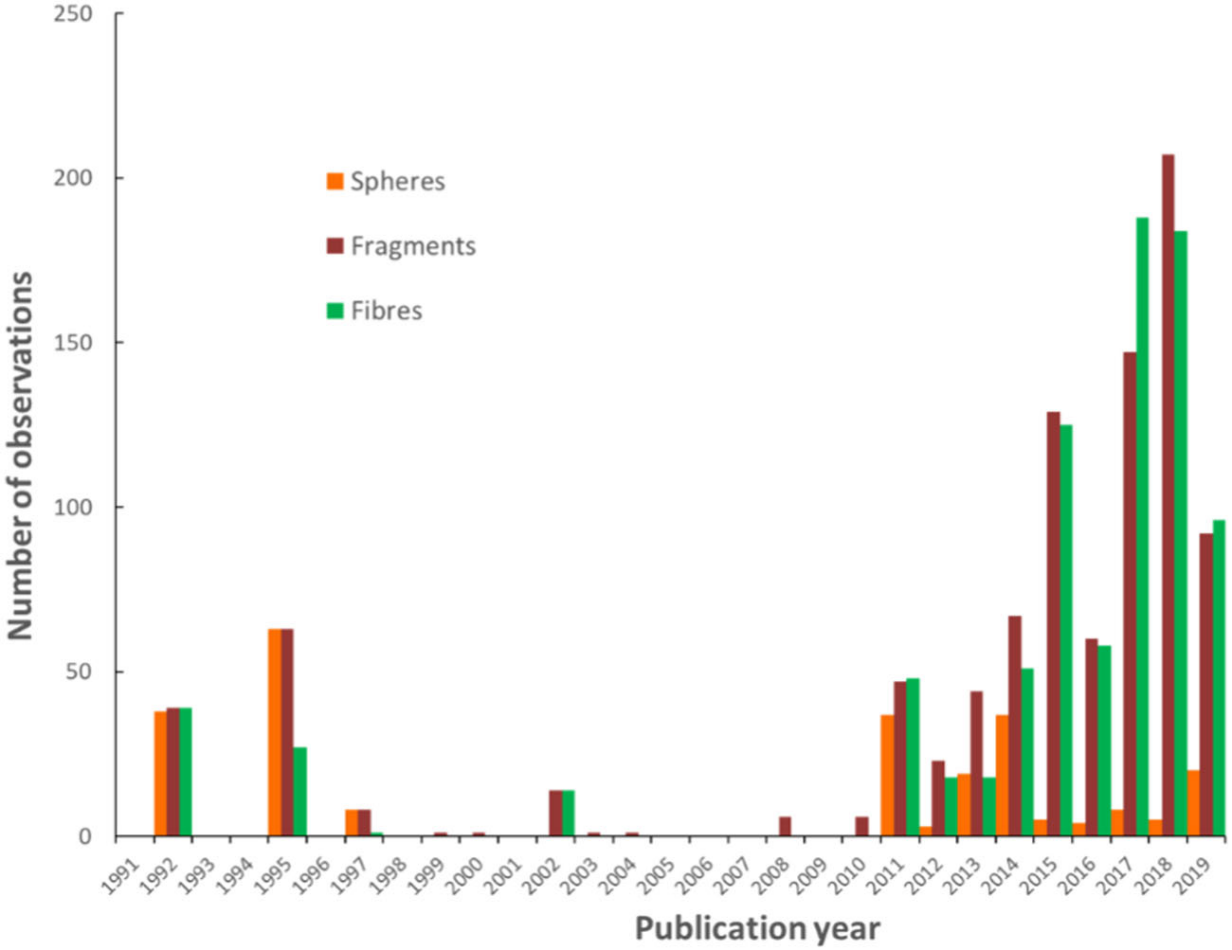
Summary of particle shapes for studies reporting on the characteristics of microplastic particle shapes ingested by biological organisms over the last 30 yr.

Another important shift in the reported characteristics of microplastic particles is a relative increase in the number of studies describing fibers as opposed to fragments and spheres (Figure 4). Early studies tended to report the ingestion of preproduction plastic spherical pellets, nurdles, or nibs (Carpenter et al. 1972; Kartar et al. 1973; Morris and Hamilton 1974; Bourne and Imber 1982; Harper and Fowler 1987). Recent studies have more frequently noted fragments and fibers. Is the reduction in frequency of preproduction plastic pellets an indication of changes in the nature of plastic debris contaminating the environment? For instance, following industry‐led initiatives to reduce the leakage of pellets to the Severn Estuary in the United Kingdom, Kartar et al. (1976) reported a decline in the ingestion of spherical pellets by fish between samples collected in summer and autumn 1973 and those collected throughout 1974/1975 (Kartar et al. 1973, 1976). The relatively rapid change in ingestion of microplastic particles is perhaps reflective of the response time of a system to clear contaminants in the surrounding environment, a concept that might be useful in providing an indication of the potential of mitigation efforts in reducing plastic litter via strategically directed biomonitoring campaigns. In this instance the response time of a system to clear contaminants refers to a shift in environmental exposure to microplastic particles. The particles may have simply been transported out of the system following advective transport processes or they may have been entrained/buried in sediment; in addition, such a decline may have been partly the result of manual removal by individuals as a result of a targeted clean‐up program. Processes such as fragmentation and settling have been suggested as potentially fundamental in explaining the rapid removal of buoyant plastic debris from the ocean surface layer; increasing settling rates have been shown to be correlated with biofilm formation and decreasing particle size following fragmentation (Koelmans et al. 2017). Regardless of which mechanism influences the removal of microplastic particles from surface waters, biomonitoring may possibly provide an effective tool to characterize the rate at which an environmental system responds to reductions in emissions of the particles; for example, biomonitoring may be able to chart the relative efficacy of the Microbead‐Free Waters Act introduced in the United States in 2015 and the associated initiatives taken by industry to voluntarily remove microbeads from their products.

Alternatively, recent awareness of the release into the environment of plastic fibers originating from textiles (Browne et al. 2010) may represent an important factor regarding an increase in the number of studies reporting on particle fibers, as illustrated in Figure 4. For instance, a recent study that reported microplastic particles in *Argonauta nouryi*, an octopod that inhabits the holopelagic zone off the Pacific coast of Southern Mexico, suggested that elevated numbers of fibers may reflect an increase in the concentration of buoyant fibers in surface waters (Alejo‐Plata et al. 2019). In this instance, Alejo‐Plata et al. (2019) reported concentrations as high as 914 fibers in a single Noury's argonaut; fibers were further observed in the stomach contents of predators consuming *A. nouryi*, implying trophic transfer. However, the polymer composition of the fibers in *A. nouryi* was not reported, and 72% of the fibers were noted to have a characteristic hyaline color. The observation of transparent hyaline fibers could imply a natural source, because this characteristic is often associated with biological tissues such as cartilage and other connective tissue fibers (Elder and Owen 1967; Bone et al. 1981; Cole and Hall 2004). Consequently, in the absence of verification of polymer composition, caution should be used when one is attempting to interpret the data reported for *A. nouryi* within the context of bioaccumulation. Indeed, the number of studies that have observed microplastic particle fibers within biological organisms in the absence of verification is significant; only approximately 35% of studies reporting fibers provided appropriate analytical verification.

Efforts to address both the potential for airborne contamination of fibers throughout sampling and laboratory preparation and the need to verify polymer composition are critical when data on microplastic particles are used to assess the extent of ingestion. Foekema et al. (2013) analyzed fish taken from the North Sea and noted considerable airborne contamination of fibers that had occurred during sample collection and laboratory analysis. Their observation subsequently resulted in recommendations for adopting a robust quality assurance/quality control (QA/QC) protocol to reduce, assess, and characterize the extent of contamination that may be present. When the method used was strengthened to reduce and appropriately account for the extent of contamination, the concentration of microplastic particles in fish was reported to be approximately 1 particle/fish, in the gastrointestinal tract (Foekema et al. 2013). More recently, Hermsen et al. (2017) also investigated the extent of microplastic particle contamination in North Sea fish using a strict QA/QC protocol and observed only 2 plastic particles in the gastrointestinal tract of 1/400 fish.

Given the lack of standardized methods available and the extent of variability regarding the application of methods used to extract, isolate, and identify microplastic particles in organisms, tools that can help guide future studies to produce more robust data are needed to better understand the extent of particle ingestion. As an initial step toward addressing this need, Hermsen et al. (2018) have articulated a scoring system that could be adopted by individual groups to help strengthen the overall quality of data produced from studies assessing ingestion.

With the increase in the number of studies reporting fibrous particles also comes a need to accurately characterize their polymeric composition, particularly if natural cellulosic fibers and/or semisynthetic fibers such as rayon are to be differentiated from synthetic plastic fibers (Remy et al. 2015; Comnea‐Stancu et al. 2017). In some instances in which fibers have been reported as the dominant shape, verification has been included and polymer composition reported (e.g., Lusher et al. 2013; Bessa et al. 2018). However, not all research groups have the analytical ability to verify polymer composition, particularly for fibers, which represent a challenging group of materials, as described by Comnea‐Stancu et al. (2017). Nevertheless, even in the absence of verification, the dominance of microplastic particle fibers in samples has been reported (e.g., Lusher et al. 2013; Bellas et al. 2016; Naidoo et al. 2016).

The inherent inconsistency in how data are analyzed and reported unfortunately creates confusion with respect to information used to help inform the decision‐making process and further limits the ability to perform detailed meta‐analyses of the data. Even when polymer composition is reported, many studies will include semisynthetic nonplastic fibers such as rayon in the total microplastic particle count (Lusher et al. 2013; Carreras‐Colom et al. 2018; Li et al. 2018; Markic et al. 2018), or they will acknowledge the challenge associated with distinguishing between natural and semisynthetic fibers, and not include them (Lusher et al. 2015). It should also be noted that in some instances fibers are not reported at all because of their observation in laboratory procedural blanks (Goldstein and Goodwin 2013; De Witte et al. 2014; Avio et al. 2015; Rummel et al. 2016; Ory et al. 2017, 2018a; McNeish et al. 2018). Overall, the characterization of microplastic particle fibers, the inconsistencies with respect to QA/QC, and the lack of harmonized analytical methods represent a nontrivial challenge for differentiating between naturally occurring and plastic fibers (Comnea‐Stancu et al. 2017). With the increasing awareness that the dominant type of anthropogenic fibers is natural nonplastic in origin, such as cotton and wool, extreme caution will continue to be needed when one is assessing their ingestion, at least until harmonized approaches for characterizing and quantifying fibers are formally adopted (Kroon et al. 2018; Stanton et al. 2019).

**Figure 5:**
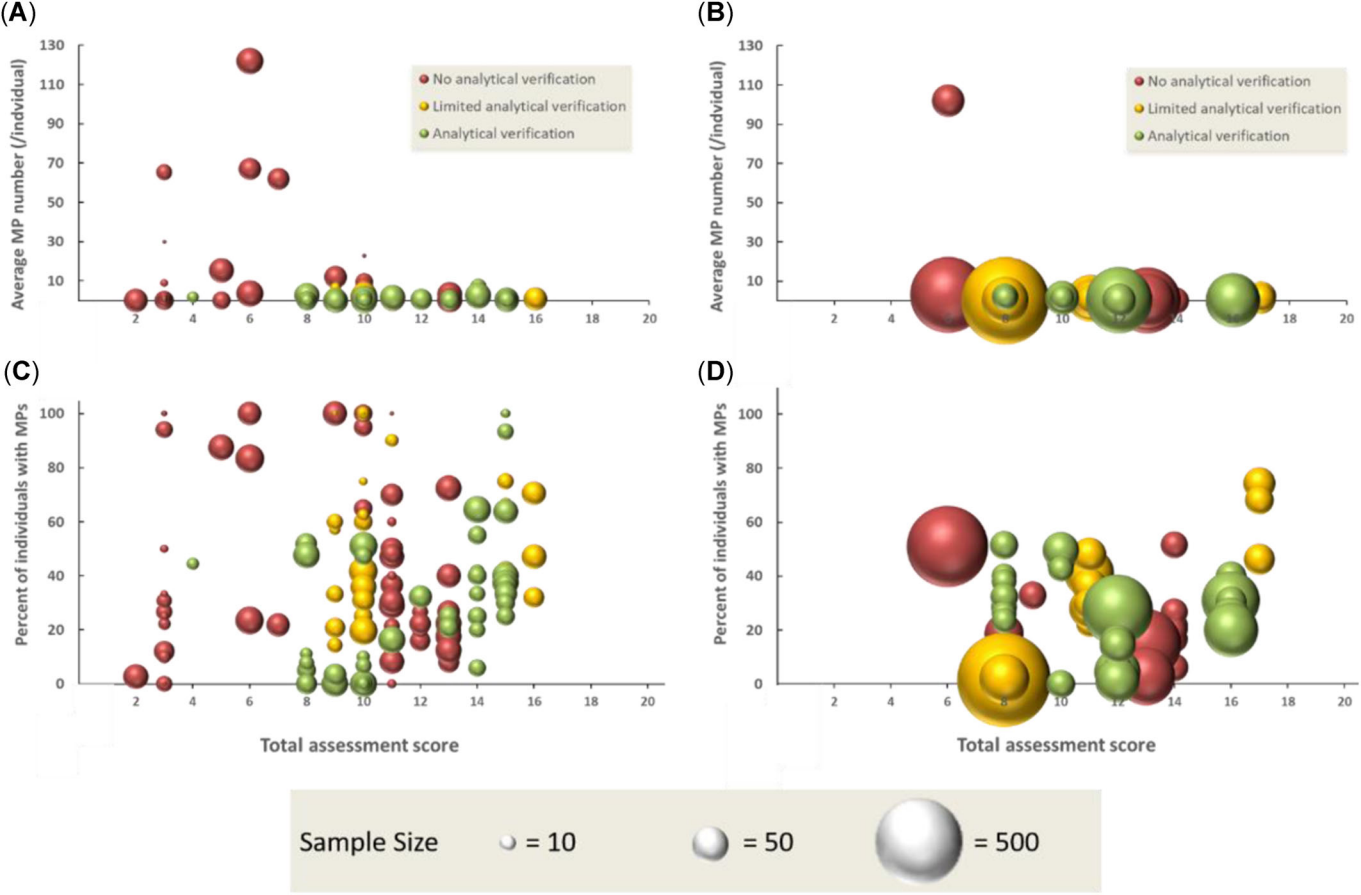
Relationship between the total assessment score and studies that reported on (**A**) average number of microplastic particles/individual with sample sizes <50, (**B**) sample sizes >50, (**C**) frequency of occurrence of microplastic in organisms with sample sizes <50, and (**D**) with sample sizes >50. Also illustrated is an indication of studies that included some level of analytical verification based on the scoring system defined by Hermsen et al. (2018); no analytical verification is indicated by red, limited analytical verification by yellow, and full verification by green. The relative sample size of each study is also illustrated, scaled to the largest sample size included in the analysis (*n* = 734). MP = microplastic.

Taking into account those studies that included analytical verification versus those that did not, Figure 5 illustrates the relationships between the TASs (using the approach described by Hermsen et al. 2018) for studies that reported both the average number of microplastic particles/individual and the percentage fraction of microplastic particles reported for individuals for a specific species within a study. The data in Figure 5 further highlight how inclusion of analytical verification may potentially influence the detection and reporting of microplastic particles, while also emphasizing inconsistencies related to sample sizes used in assessing the extent of ingestion.

In Figure 5A,B the average number of microplastic particles/individual is illustrated in relation to the overall TAS and to the level of analytical verification included in the study. For the data included in Figure 5, 40% of studies included analytical verification and 40% did not; the remaining 20% of studies provided limited verification. Whereas most studies reported an average microplastic particles/individual of between 0 and 10, a few studies reported notably much higher values. For instance, the data reporting averages of >60 microplastic particles/individual are from the study on *A. nouryi* of Aleja‐Plata (2019), and as discussed just above, verification of the fibers dominating the total average was not conducted. Nevertheless, the values reported by Aleja‐Plata (2019) are consistent with the average value of 57.2 microplastic particles/individual reported for *Patinopecten yessoensis* by Li et al. (2015), who did perform verification. However, the elevated levels of fibers in *P. yessoensis* and other farmed mussels collected from a Chinese fish market, compared with wild mussels were reported to be due to direct ingestion of plastic fibers from the polypropylene lines on which the mussels are grown (Li et al. 2015).

In Figure 5 the data symbols are scaled to the sample size (normalized to the largest data point in the plot, which is *n* = 734), and which represents an additional metric included in the TAS. In Figure 5A,B the relative sample size does not appear to influence the average microplastic particle number/individual reported, with both high and low sample sizes generally resulting in values ranging from 0 to 10 particles/individual. In this instance, analytical verification may be more important in determining the number of particles ingested by an individual.

Figure 5C illustrates the relationship between the TAS and the percentage fraction of microplastic particles ingested by individuals of a species for samples with <50 individuals, and Figure 5D illustrates the same relationship for samples with >50 individuals. Several important points can be made from Figure 5C,D. For instance, the influence of sample size in relation to the frequency of detecting microplastic particles within a population of a species appears to be an important parameter in establishing the propensity of a species to ingest microplastic particles. In studies that reported a frequency of detection >60%, the sample sizes were predominantly <50. However, where frequency of detection is <50%, there is also considerable scatter in the data, making it difficult to interpret any statistically significant relationships. Intuitively, larger samples sizes should provide stronger evidence establishing both the extent to which microplastic particles are contaminating the external environment and the propensity for a species to ingest plastic debris. It is therefore notable that the studies tending to report high frequency of detection were generally based on relatively small datasets, with lower frequency of detection more strongly associated with larger sample sizes (Figure 5D).

For instance, data for 2 fish species included in Figure 5C show a frequency of occurrence of >90%, supported by analytical verification. The 2 fish species are juvenile coral trout (*Plectropomus leopardus* and *P. maculatus*; Kroon et al. 2018). The fish were collected in the waters of the Great Barrier Reef (Australia), and anthropogenic fibers represented by synthetic, semisynthetic, and naturally derived polymers were detected. The authors suggest that analytical verification represents an important component in our understanding of microdebris ingestion by fish in that the ability to differentiate between naturally derived and synthetic polymeric particles can help us better assess potential sources of contamination (Kroon et al. 2018). However, these authors’ sample sizes were relatively low (*n* < 10), so extrapolation of the relative propensity of the species for ingesting microplastic particles would be of concern. Future research would benefit from a greater mechanistic understanding of physiological and behavioral traits that may cause some species to ingest the particles to a greater extent than others.

Characterizing and quantifying the propensity for an organism to ingest microplastic particles can provide useful information in identifying species that may have physiological and behavioral traits making them susceptible to bioaccumulation of microplastic particles; for example, birds might ingest gravel to help grind hard seeds or bones in the gizzard (Day 1980; Reid 1981; Azzarello and van Vleet 1987; Kiorboe 2011; Carson 2013; Kiorboe and Hirst 2014; Schuyler 2014; Deudero and Alomar 2015; Peters and Bratton 2016; Ryan 2016; Mizraji et al. 2017; Fossi et al. 2018; Battisti et al. 2019; Franzellitti et al. 2019). In these instances, the relative size of the sample collected likely represents an important factor for assessing relationships between frequency of occurrence and key species‐specific traits.

Generally, the data presented in Figure 5 provide an illustrative representation of the extent of the inconsistency and heterogeneity in how data have been developed thus far, which greatly complicates working toward greater mechanistic understanding. Consequently, a key message from Figure 5 is that greater confidence in the interpretation of data reporting the ingestion of microplastic particles in organisms will undoubtedly result in higher weighting of those studies providing analytical verification versus those that do not; in addition, where possible, sample sizes sufficient to be statistically robust would provide added value. For instance, both the International Council for the Exploration of the Sea (OSPAR Commission 2015) and the European Marine Strategy Framework Directive Technical Subgroup on Marine Litter (2013) recommend a sample size of at least 50 individuals, with Hermsen et al. (2018) using this suggestion to attribute higher scores to samples sizes >50. Thus, interpretation of data from past studies reporting elevated levels of fibers and other fragments and spheres that have been qualitatively defined as microplastic particles in the absence of analytical verification, and which are based on sample sizes <50, represents substantive challenges when one is attempting to compare them with more recent studies of higher quality. The inherent inconsistencies in how data are collected and reported and the associated challenges in data interpretation should thus not be underestimated when one is working toward the development of a quantitative weight‐of‐evidence understanding that can help support, refute, and/or mechanistically assess the potential of a species to ingest and potentially bioaccumulate microplastic particles.

For instance, Lefebvre et al. (2019) found that only a small fraction of all debris ingested by sardines (2.3%) and anchovies (1.5%) could be identified as microplastic particles, results that are consistent with other studies also reporting that natural fibers dominate the composition of particles ingested (Remy et al. 2015; Zhao et al. 2016; Catarino et al. 2018; Kroon et al. 2018). Misidentification is not necessarily isolated to fibers but can also include spheres. For instance, Li et al. (2015) observed a large number of uniform transparent spheres in the bivalve *Scapharca subcrenata* that were determined to be aluminum silicate when analytically assessed by FTIR. The weight‐of‐evidence from these studies would imply that ingestion of both natural and synthetic particulates is common, information that might be used to help clarify the strategies species have evolved in relation to particulate exposure. An increased understanding of this issue could thus be used in developing methods to assess ecotoxicological risks from emissions of anthropogenically derived particles, both synthetic and naturally derived.

An obvious benefit of using analytical tools to verify polymeric composition of particles observed to be ingested by organisms is the ability to report polymer type and to differentiate between natural and synthetic polymers. In the studies included in the present review that have reported polymeric composition, the microplastic particles observed tended to be dominated by polyethylene, polypropylene, polystyrene, polyvinyl chloride, and polyethylene terephthalate, which are typically associated with plastic fragments, pellets, or films (see the Supplemental Data). The polymeric composition of fibers, which represent a significant group of materials (Figure 4), tends to be dominated by polyamide, polyester, acrylic, polyethylene terephthalate, polyvinyl chloride, polyethylene, and polypropylene. In addition to synthetic polymeric materials, semisynthetic fibers such as rayon/viscose are often reported (as previously discussed in this section), as well as natural fibers of cotton and wool and other unidentified natural cellulosic materials and proteins. Occasionally, there are reports of other synthetic polymers, such as ethylene vinyl acetate, styrene acrylate, polyurethane foam, polybutylene terephthalate, polymethyl methacrylate, and polytetrafluoroethylene (Teflon). When polymeric verification was not included, studies have often reported on the characteristics of larger fragments of plastic, typically differentiating particles associated with consumer or industrial plastic, and have included descriptions such as plastic bags, rubber elastics, rubber balloons, cigarette holders, food packaging, and so on. An overall trend is that the ingestion of microplastic particles and other debris is typically reflective of the litter in the local area in which the organisms live (Joyce et al. 2002; Anastasopoulou et al. 2013; Wojcik‐Fudalewska et al. 2016; Zhao et al. 2016; Erni‐Cassola et al. 2019; Lefebvre et al. 2019).

Consistent with recommendations from other studies, the ingestion of microplastic particles by specific organisms may be useful as part of a biomonitoring program aimed at prioritizing mitigation actions to reduce the release of polymers and product use scenarios that contribute to anthropogenic debris observed in the environment (OSPAR Commission 2008; Avery‐Gomm et al. 2012; European Commission 2013; Bellas et al. 2016; Bessa et al. 2019; Bray et al. 2019). For instance, in Europe, the Marine Strategy Framework Directive has established an aim of assessing progress toward the achievement of Good Environmental Status for European waters. This requires all member states to ensure that “the amount of litter and micro‐litter ingested by marine animals is at a level that does not adversely affect the health of the species concerned” (European Commission 2013). The proposed threshold used under the Marine Strategy Framework Directive to quantify the level of litter being ingested is largely based on recommendations from the Convention for the Protection of the Marine Environment of the North‐East Atlantic (the OSPAR Convention). Under OSPAR an example of a threshold value for assessing Good Environmental Status would be based on monitoring the quantity and incidence of litter ingested by *Fulmarus glacialis*, North Sea northern fulmars, where a quantitative level target of <10% of North Sea fulmars should have no more than 0.1 g of plastic in their stomach over a continuous period of at least 5 yr in all North Sea regions (OSPAR Commission 2008; European Commission 2013). Generally, the stomach contents of northern fulmars have proved to be a cost‐effective biomonitor and can provide timely information related to the effectiveness of mitigation efforts at reducing plastic pollution by assessing both increasing and decreasing trends (van Franeker et al. 2011; Avery‐Gomm et al. 2012; Trevail et al. 2015; Beer et al. 2018).

Building on the use of northern fulmars as a biomonitor for the North‐East Atlantic and North Sea, there have been additional efforts to identify other species that may lend themselves to biomonitoring. A key challenge is to identify species with traits and spatial distributions that may make them suitable. For instance, in assessing the suitability of nearly 50 different fish species to act as biomonitors for the Mediterranean basin, Bray et al. (2019) have suggested *Engraulis encrasicolus*, *Boops boops*, and 3 species of Myctophidae (*Hygophum benoiti*, *Myctophum punctatum*, and *Electrona risso*) as well as *Mullus barbatus barbatus* and *Chelidonichthys lucerna* as suitable pelagic, benthopelagic, mesopelagic, demersal, and benthic biomonitoring species, respectively.

### Assessment scores

**Figure 6:**
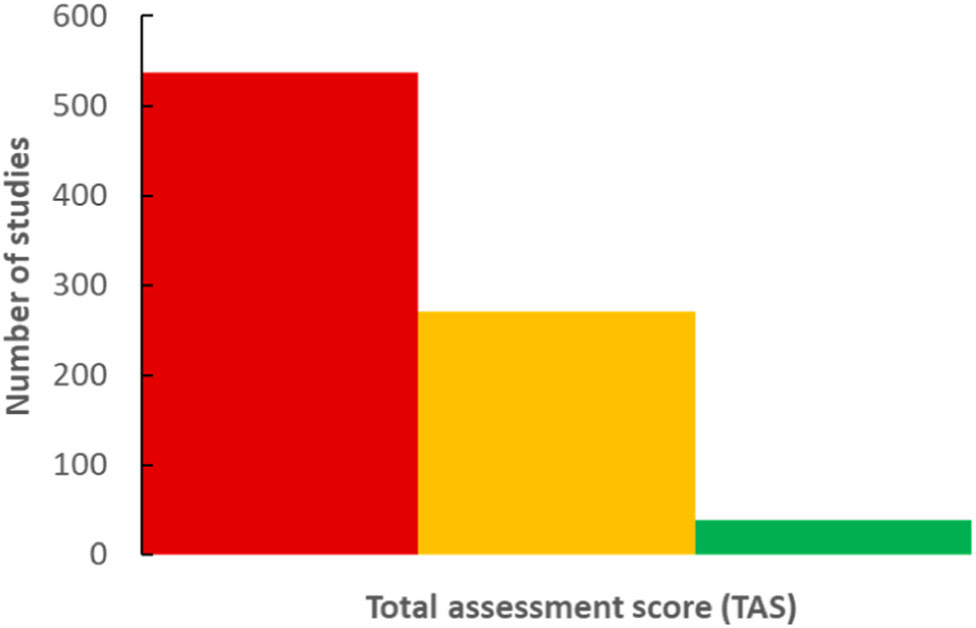
Summary of total assessment scores (TASs) from 851 field‐based observations: TAS <10 categorized as poor quality (red); TAS >10 but <15 categorized as medium quality (yellow); and TAS >15 categorized as high quality (green). The scoring system is based on that developed by Hermsen et al. (2018).

Although the use of standardized indicator species will likely help strengthen the consistency of how data are reported (making data more comparable for assessing both spatial and temporal trends), the complementary application of a standardized analytical protocol will also be required. Recognizing the lack of consistency in how samples are collected, stored, handled, processed, and analyzed, Hermsen et al. (2018) have developed a scoring system that allows assessment of the overall quality of a particular study. Figure 6 illustrates the distribution of the TAS for 129 publications representing 851 separate observations from field‐based studies that were assessed as part of the present review.

The studies assessed largely represent those published since 2010. Prior to 2010, methods for extracting and analyzing debris specifically for microplastic particles were largely absent. Subsequently, it can be assumed that studies prior to 2010 will largely have TAS <10. As confirmation, one study from 1970 reporting on the stomach contents of *Alepisaurus ferox* (lancetfish) by Kubota and Uyeno (1970) was included; these authors identified 78 pieces of plastic in 36 fish, as well as pieces of wood and bamboo, and the skin of an onion. The TAS from this study is 3, but the results are nonetheless perceived as useful in that the stomach contents reported appear to be consistent with debris that was present in Suruga Bay, Japan, where the fish were sampled, an observation that is generally consistent among all field‐based studies reviewed, particularly over the last 50 yr (Kubota and Uyeno 1970). As a contrast to the observations of Kubota and Uyeno (1970), another early study from 2002 (Cliff et al. 2002), which included the analysis of 28 687 sharks between 1978 and 2000, was also assessed based on the criteria of Hermsen et al. (2018). The TAS is 5 for the study of Cliff et al. (2002), but, as with the study of Kubota and Uyeno (1970), the observations reported, particularly for such a large dataset and time frame, are useful in that only 0.38% of sharks contained plastic debris, which was largely identified as plastic bags, sheets, food packaging, and ropes. No temporal trend was reported, and the data may thus be useful for establishing baseline information. The analyses of both Kubota and Uyeno (1970) and Cliff et al. (2002) largely focused on larger plastic particles (>1 mm), items that could be readily identified without the aid of visual magnification, a factor that strongly influenced the low scoring for these 2 studies for the purposes of assessing microplastic particle ingestion, which is typical of studies prior to 2010.

Following the development and application of extraction and isolation methods, such as for *F. glacialis* (van Franeker et al. 2011), mesopelagic fish (Davison and Asch 2011), decapod crustaceans (Murray and Cowie 2011), and other organisms such as mussels (Claessens et al. 2013; Van Cauwenberghe and Janssen 2014), the ability to measure microplastic particles ingested by biological organisms has greatly improved. However, the methods used are not necessarily representative of a standardized protocol; there is large variability with respect to how samples are collected, stored, and extracted. There is also inconsistency with respect to digestion methods; both acid‐ and alkaline‐based techniques (and more recently enzymatic methods) are employed, and there is substantial variation in the pore sizes of the filter membranes used to filter digestate. These and other analytical challenges, which can strongly influence the size distribution of microplastic particles that can be reported, are generally well understood and have been thoroughly reviewed in various recent publications, in which a need to develop standardized analytical methods is generally advocated (Rocha‐Santos and Duarte 2015; Courtene‐Jones et al. 2017; Lusher et al. 2017; Hermsen et al. 2018; Bessa et al. 2019; Claro et al. 2019).

Such inconsistencies are highlighted in Figure 6, where most studies can be seen to have a TAS ≤10, based on the scoring criteria defined by Hermsen et al. (2018). No relationship exists that might demonstrate an improvement in TAS for more recent publications, and thus the overall quality of data being produced continues to represent cause for concern. In most instances the criteria that tend to influence TAS ≤10 are those related to the QA/QC protocol. For instance, more than half of the studies with a TAS ≤10 were assigned a score of 0 for failing to include measures to reduce laboratory contamination (57%); failing to process samples within a laminar flow fume hood cupboard or dedicated clean‐air laboratory (90%); not including proper use of negative controls (64%), such as field, travel, and laboratory blanks processed at the same time as sample batches; failing to include positive controls (91%) such as reference materials to assess the recovery efficiency of the digestion method; not including a sample preparation method (60%) such as acid or alkaline digestion, but instead isolating microplastic particles under a microscope using manual techniques; and finally, failing to analytically verify that the particles identified are indeed composed of synthetic polymers (60%). For studies with 10≥ TAS ≤15, certain QA/QC protocols also tend to be poorly adhered to, specifically the satisfactory use of negative and positive controls and the processing of samples in a dedicated clean‐air laboratory or under a laminar flow fume hood. Given the relatively low numbers of microplastic particles/individual typically reported, strict adherence to a QA/QC protocol must be key in any future study aimed at quantifying the ingestion of microplastic particles, particularly for the purposes of assessing the potential for biological uptake and/or trophic transfer. Studies with TAS >15 largely address all criteria, and the data reported can be used to apply quantitative weight‐of‐evidence approaches for assessing and interpreting the implications associated with the ingestion of microplastic particles.

### Bioaccumulation and trophic transfer of microplastic particles

With an awareness of the substantial heterogeneity associated with the reported data, it may at least be possible to qualitatively consider the various lines‐of‐evidence that support or refute the potential for bioaccumulation and trophic transfer resulting in the biomagnification of microplastic particles. As noted previously in the *Introduction*, bioaccumulation defines a process by which a contaminant (bio)accumulates within the tissues of an organism to a level that is greater than that of the surrounding environment (Gobas and Morrison 2000). The overall mass or number of microplastic particles accumulated in the tissues of an organism would thus need to greatly exceed the egestion of microplastic particles, which would result in a mass balance demonstrative of a quantitative potential for (bio)accumulation. An understanding of species‐specific ingestion and egestion rates is thus needed to quantify and mechanistically assess the overall fate of the particles with respect to biological uptake and potential for trophic magnification (Diepens and Koelmans, 2018). However, when the data were reviewed, it could be seen that the location of the particles within the organism was predominantly in the stomach or gastrointestinal tract. Approximately 90% of all field‐ and laboratory‐based studies found that the microplastic particles were isolated within the stomach or gastrointestinal tract and/or excreted with feces. These observations included both vertebrates and invertebrates, with the latter samples occasionally pooled or analyzed as whole tissue, preventing a determination of where within the organism the particles were located. The high frequency of detection in the gastrointestinal tract was largely because tissue analysis was generally limited to the gastrointestinal tract, based on the underlying assumption that the particles are too large to readily cross epithelial tissues.

Nevertheless, several laboratory‐based studies have reported translocation of microplastic particles, mostly nanosized plastic, from the gastrointestinal tract into other tissues (Browne et al. 2008; Rosenkranz et al. 2009; von Moos et al. 2012; Farrell and Nelson 2013; Avio et al. 2015; Brennecke et al. 2015; Mattsson et al. 2017; Skjolding et al. 2017; Al‐Sid‐Cheikh et al. 2018; Chae et al. 2018; Ding et al. 2018; Pitt et al. 2018; Triebskorn et al. 2019), implying that when exposure to microplastic particles of a particular size is elevated, translocation from the gastrointestinal tract to internal organs is possible. In some studies, particles coated with a fluorescence dye were used to visually observe the behavior of particles, such as in an early study by Rosenkranz et al. (2009). However, as recently discussed by Schur et al. (2019), caution may be warranted not to overinterpret the potential for translocation based on observations utilizing fluorescence, because results may be characteristic of a study artifact (i.e., lipid accumulation of the leached fraction of hydrophobic fluorescent dye), as opposed to actual particle translocation. Schur et al. (2019) attempted to replicate earlier observations, but a general lack of reproducibility of studies that found translocation has been cited as cause for concern (Burns and Boxall, 2018). This is an important cautionary note, in that recent publications (Schur et al. 2019; Triebskorn et al. 2019) have drawn attention to the inherent challenges that particles face when crossing cellular membranes. Consequently, the ability to replicate results is important in working toward a greater mechanistic understanding of the biological uptake of particles.

In daphnids, for instance, particles must cross the peritrophic membrane prior to translocation across the epithelium of the digestive tract and transportation to a target tissue. As has been noted (Schur et al. 2019; Triebskorn et al. 2019), the peritrophic membrane plays an important role in preventing the translocation of particles; it has evolved to prevent mechanical damage due to the ingestion of naturally occurring particles and to block the biological uptake of toxic pathogens. Due to analytical limitations, however, existing data from field‐based studies are unable to support or refute the extent to which translocation of nanoplastics might occur under environmentally relevant conditions; this may represent an area for further study. Given the research that has been conducted into the bioaccumulation of ENMs, considerable knowledge may already be accessible, and it is recommended that opportunities to build on currently available information should be optimized (Hüffer et al. 2017). For instance, ^14^C‐labeled polystyrene nanoplastic has recently been employed to study the uptake and depuration of nanosized particles at environmentally relevant concentrations (Al‐Sid‐Cheikh et al. 2018). Al‐Sid‐Cheikh et al. (2018) observed relatively rapid depuration of the particles, but proposed further research using ^14^C‐labeled particles aimed at better characterizing and quantifying the mechanisms of uptake, absorption, and elimination of particles in chronic exposures, which may help us to better understand the fate of biologically ingested particles.

An important observation in the context of assessing the potential of microplastic particles to bioaccumulate is therefore the need to demonstrate accumulation of the particles within the internal tissues of the organism. Does detection of particles in the gastrointestinal tract imply biological uptake? The contents of the gastrointestinal tract are typically not considered to be internalized by an organism (DeVito 2000; Gobas and Morrison 2000; Kenyon and Hughes 2010) and would thus be considered present externally. Therefore, it is currently unclear how the majority of field‐based studies, which focus on analyzing microplastic particles in the stomach and gastrointestinal tract of the organism, can be used in interpreting the potential of the particles to bioconcentrate and biomagnify. Depending on the species under investigation and the presence or absence of food, the residence time in the gastrointestinal tract ranges from minutes to days; the particles themselves can be considered to be simply passing through, and the data illustrative of snapshots in space and time.

This understanding is consistent with results reported in various laboratory‐based studies, which have often found efficient egestion of microplastic particles (Graham and Thompson 2009; Cole et al. 2013; Ugolini et al. 2013; Chua et al. 2014; Kaposi et al. 2014; Hu et al. 2016; Ogonowski et al. 2016; Grigorakis et al. 2017; Dawson et al. 2018; Ory et al. 2018b; Woods et al. 2018; Fernandez and Albentosa 2019; Song et al. 2019). Efforts directed toward further characterization of gastrointestinal tract residence times would thus help to illuminate an important parameter in developing mass balance models aimed at assessing ingestion and trophic transfer and would further increase our understanding of the potential of the particles to bioaccumulate (Al‐Sid‐Cheikh et al. 2018; Diepens and Koelmans 2018). For instance, the rate of uptake across the epithelium tissues of the gastrointestinal tract would need to be considered in relation to the relative concentrations of particles in the gastrointestinal tract and their residence time. Under environmentally relevant concentrations, where concentrations in the gastrointestinal tract are not at steady state with the external environment, and where the rate of passage through the gastrointestinal tract may be greater than the rate of biological uptake, accumulation within the organism is unlikely. Alternatively, when the gastrointestinal tract residence time is relatively long compared with the rate of biological uptake, then an increased probability for translocation may be possible (such as observed in the theoretical MICROWEB model applied to seals by Diepens and Koelmans [2018] and to fish by Roch et al. [2020]). It is thus recommended that future studies should include efforts aimed at estimating gastrointestinal tract residence times for the species under investigation; such studies should also aim to characterize potential differences between species with different feeding strategies (i.e., filter‐feeders vs nonfilter‐feeders). Such insights would help to strengthen our mechanistic understanding of ingestion and the potential for biological uptake of microplastic particles.

**Figure 7:**
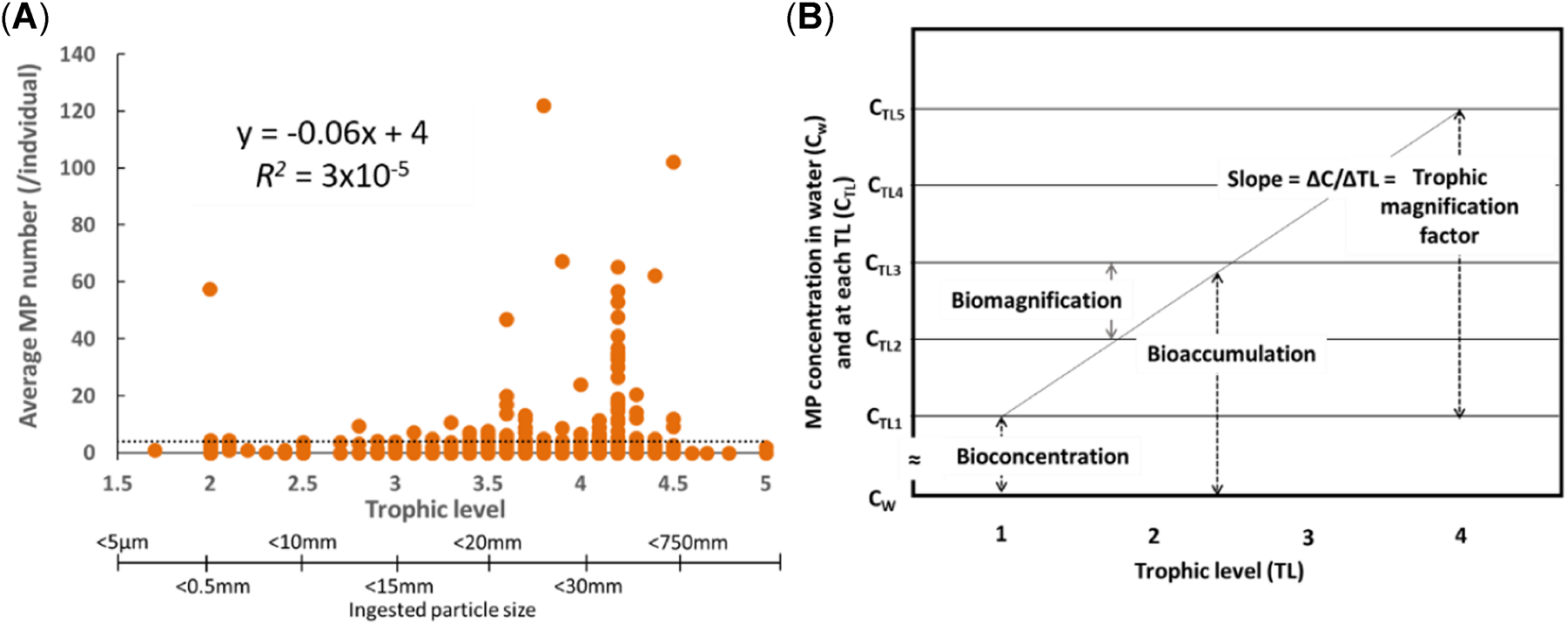
Relationship between (**A**) average number of microplastic particles (MP) ingested/individual and their relative trophic level (TL; *n* = 517) and (**B**) the various bioaccumulation metrics, where bioconcentration reflects an increase in the organism concentration relative to the water concentration; biomagnification reflects an increase in organism concentration from one trophic level to higher levels; bioaccumulation reflects an increase in organism concentration relative to both the water concentration and dietary concentration; and trophic magnification factor reflects the relative change in organism concentration across a number of trophic levels (modified from Mackay et al. 2013). It is notable that as trophic level increases, the particle size distribution of particles ingested by an organism also changes, as does the efficiency of particle egestion. Average microplastic particle concentration across all data is 4 particles/individual (dotted line) and typically ranges between 0 and 10 particles/individual. The elevated numbers illustrated for trophic level 4.2 are entirely based on results reported for *Fulmarus glacialis*, representing the species with the highest propensity for ingesting plastic debris and microplastic particles.

To characterize gastrointestinal tract residence times, it will be necessary to consider the influence of particle size, an important parameter with respect to defining the size distribution a specific species can ingest and subsequently egest. The data under scrutiny in the present review appear to show a general relationship between the relative trophic level of an organism and the average size particle that can be ingested (Figure 7). Whereas the potential to ingest micro‐ and nanosized particles exists for organisms at various trophic levels, there is intuitively an upper limit regarding what a species can egest. All organisms will have evolved mechanisms to inhibit the translocation of small particles as well as strategies related to particle ingestion (Schultz 1976; Gophen and Geller 1984; Hart 1991; Riisgård and Larsen 2010; Barua and Mitragotri 2014; Vinther et al. 2014; Schur et al. 2019; Triebskorn et al. 2019). An understanding of the particle size distribution that a species ingests and the relative propensity of a species to ingest can clarify how data from a species might be used. For instance, the development of an intelligent monitoring strategy (to determine the organisms with a propensity to ingest a wide range of particles as well as prey that may also have consumed microplastic particles) may provide useful information with respect to the relative level of bioavailable debris and the extent of trophic transfer. In consideration of the relative sample sizes required to evaluate both spatial and temporal trends and the fact that microplastic particles are typically limited to the gastrointestinal tract, it may be beneficial to consider a monitoring strategy that utilizes commercial fish.

In the collated dataset from the present review (see the Supplemental Data), a variety of commercial fish are represented. Using the bioindicator index proposed by Bray et al. (2019), 40 commercial fish species were evaluated for their potential to act as biomonitors on a global scale (see the Supplemental Data). Several fish species were found to have a bioindicator index >3, including lancetfish, mullet, scad, plaice, mackerel, seabream, and flounder. Key parameters of the bioindicator index are the presence of microplastic particles at a level generally >20%, global distribution of the species, and the fact that the species does not travel large migratory distances. Given the commercial value of the fish species, it is thus suggested that strategic collection of the gastrointestinal tract of individuals be performed, aimed at developing a consistent dataset for use in evaluating both global and regional temporal and spatial trends. Such a program would be analogous to the use of northern fulmars to assess Good Environmental Status as defined within the Marine Strategy Framework Directive.

Various concerns have been raised regarding the presence of microplastic particles within the gastrointestinal tract of an organism. For instance, the physical presence of particles may result in blockage of the gastrointestinal tract, which can negatively impact growth and reproduction and/or mortality (Gophen and Geller 1984; Wright et al. 2013; Pedá et al. 2016; Barbanera 2018; Franzellitti et al. 2019). It has also been suggested that the accumulation of both macroplastic and microplastic particles in the stomach and/or gastrointestinal tract can lead the organism to feel satiated, resulting in decreased feeding, which will also negatively impact growth and reproduction and mortality (Thompson 2015). Negative impacts associated with the ingestion of microplastic particles have been reported from laboratory‐based studies at elevated concentrations; such impacts are not consistent with environmental exposures (Huvet et al. 2016; Lenz et al. 2016). However, under environmentally relevant exposures, the numbers of microplastic particles residing in the gastrointestinal tract of fish and invertebrates reported in the literature are not generally considered harmful (Kaposi et al. 2014; Devriese et al. 2015; Mazurais et al. 2015; Alomar et al. 2017; Ory et al. 2018b; Roman et al. 2019). It should be noted, however, that harmful effects, particularly in sea turtles and sea birds, in relation to the ingestion of macroplastics are well documented, and have resulted in death (Day 1980; Reid 1981; Balaz 1985; Plotkin and Amos 1990; Thompson 2015; Acampora et al. 2016; Rizzi et al. 2019). When all the data reporting particle concentrations were considered, the average concentration of microplastic particles across all the field‐based studies for all species included in the present review was found to be 4 particles/individual, as illustrated in Figure 7A, with actual average numbers showing significant variation, ranging from 0 to 122 particles/individual (standard deviation = 11).

Consistent with observations of Diepens and Koelmans (2018), the data collected in the present review support the argument that microplastic particles ingested by organisms at lower trophic levels can be ingested by predators at higher trophic levels, and thus the relative efficiency of egestion results in less potential for magnification through the food web. The data shown in Figure 7A support this suggestion, and also strengthen the argument that observations of particles within the gastrointestinal tract of organisms are simply snapshots in time and space. For instance, if microplastic particles were biomagnifying within the food web, then the results of Figure 7A would show a significant increase in the average number of particles/individual at increasing trophic levels, with the number of particles ingested by species at higher levels being increased due to ingestion of multiple prey (i.e., trophic transfer) and direct exposure from the environment (as conceptually illustrated in Figure 7B). However, the average number of microplastic particles/individual is limited to a relatively narrow range, between 0 and 10, with an estimated trophic magnification factor of –0.06 (derived from the slope of the regression; Figure 7A). This implies that trophic magnification is not significant. The exception in Figure 7 is trophic level 4.2, for which significant variance is observed. In this instance, the variance is entirely based on the results reported for *F. glacialis* and other seabirds, in which larger particles (1–10 mm) tend to be more prevalent, and whose egestion is less efficient. Thus, although specific species may have a higher propensity to ingest particles than others, no mechanistic basis appears to exist that would support biomagnification of microplastic particles.

The main objectives of the present review (to assess the potential of microplastic particles to bioconcentrate and biomagnify, consistent with the conceptual model shown in Figure 7B) are complicated by a variety of factors, largely related to inconsistencies in analysis and reporting. For instance, the average concentration of particles in organisms varies from 0 to 122 particle/individual. Concentrations of microplastic particles in surface waters such as lakes and rivers, on the other hand, range from 0 to 10^3^ particles/m^3^ (Koelmans et al. 2019), and concentrations in oceans range from 0 to 10^6^ particles/km^2^ (Law et al. 2014). A fundamental challenge in assessing the potential for microplastic particles to bioconcentrate and biomagnify is therefore the need for a method that accounts for both the differences in units used in reporting concentrations and the stochastic and dynamic nature inherent in the ingestion and egestion of the particles. For instance, given that the analysis of data is dominated by observations in the gastrointestinal tract of organisms, which reflects a dynamic process—whereby the particles are largely passing through the organism—how can such data be used to assess bioaccumulation?

The lack of a significant relationship (*R* ^2^ = 3 × 10^−5^; slope = −0.06) between trophic levels (Figure 7A) in the concentration of microplastic particles/individual provides a line‐of‐evidence that appears to support the suggestion that the biological ingestion of microplastic particles is representative of a dynamic process, strongly influenced by the efficiency of the egestion rate of organisms at higher trophic levels, and that is not demonstrative of accumulation. It should be noted, however, that the data are limited in that they only reflect concentrations in the gastrointestinal tract, not the tissues, which represents a knowledge gap with respect to assessing bioaccumulation and trophic transfer. Therefore, based on the available data, the weight‐of‐evidence does not support the existence of bioaccumulation for microplastic particles, but this assessment is limited to the availability of data from the biological tissues (i.e., stomach and gastrointestinal tract) where accumulation of contaminants is unlikely to occur.

## CONCLUSIONS

This section will address certain limitations of the present review and will summarize future research needs. In reviewing the data related to the ingestion of microplastic particles within the context of assessing their bioaccumulation potential, it becomes readily apparent that the existence of microplastic particles within the gastrointestinal tract of organisms is not consistent with the potential for the particles to bioaccumulate. The mechanisms of ingestion and egestion effectively result in a mass balance that does not engender internal accumulation; the particles simply pass through the organism along with its natural food. The presence of food may enhance the efficiency of egestion, and research targeting the influence of food on egestion rates would likely help in the development of mass balance models aimed at assessing the role of organisms in influencing the overall fate of microplastic particles. For instance, some studies have observed that ingestion followed by egestion of fecal pellets may represent an important transport process that facilitates the movement of buoyant particles into the deep sea and/or sediment (Long et al. 2015; Cole et al. 2016; Katija et al. 2017; Wieczorek et al. 2018, 2019). This transport concept could help explain why microplastic particles on the sea surface layer do not appear to be increasing (Law et al. 2010; Koelmans et al. 2017). Could the ingestion and egestion of microplastic particles represent an important environmental fate process that facilitates their potential to become entrained in soil and sediment systems? Or does this shift in environmental fate result in greater exposure to benthic and terrestrial organisms? Quantitative characterization of the mechanisms that influence particle cycling may prove beneficial in assessing environmental persistence as opposed to bioaccumulation. Consequently, future research should give careful consideration to problem formulation.

The studies included in the present review have sampled >87 000 individuals in an effort to assess the extent of ingestion of microplastic particles and plastic debris. Given that researchers have observed microplastic particles in approximately 20% of individuals, continuing efforts to sample novel species and higher numbers of individuals should be carefully assessed. How will observations of microplastic particles in novel species add value to the breadth of data that already exists? Similarly, will increasing sample sizes to strengthen statistical rigor add significant value? These questions should be considered from the perspective of efforts to reduce the use of animals in research, and thus they represent important moral and ethical issues. Given the variability in the development and application of analytical methods for characterizing and quantifying microplastic particles ingested by biological organisms, research activities that work toward the development of standardized methods should be given the highest priority. The availability of standard and harmonized methods would result in greater confidence in and utility of information and data obtained from future studies.

The data reporting on biological ingestion of microplastic particles thus far clearly demonstrate the potential of the particles to be ingested by organisms at all levels of biological organization. The data also imply that whenever anthropogenic debris is present and environmentally available, individuals of certain species will ingest and subsequently egest it, regardless of whether it is a synthetic plastic particle or other naturally derived anthropogenic particles. Reducing anthropogenic debris thus represents an important and aspirational goal for society to work toward. The effective ability of society to mitigate the release of debris may be efficiently achieved via well‐defined strategically implemented biomonitoring programs, which could be coordinated to address local, regional, and international regulatory drivers. The Marine Strategy Framework Directive and OSPAR Convention currently provide model systems on which to build. Thus, within the community of researchers assessing microplastic particles, consensus is greatly needed on identification of species and standardized protocols.

## Supplemental Data

The Supplemental Data are available on the Wiley Online Library at https://doi.org/10.1002/etc.4718.

## Supporting information

This article includes online‐only Supplemental Data.

Supporting information

## Data Availability

Data are available on request to the corresponding author (todd.gouin@environresearch.com). All the data for the present study are available in the Supplemental Data.
